# Environmental responsibility in the Sterile Processing Department: nursing leadership in sustainable development

**DOI:** 10.1590/0034-7167-2025-0487

**Published:** 2026-10-16

**Authors:** Renata Pereira Silva Artioli, Sidiclei Machado Carvalho, Maricel Silveira Corrêa, Denilse Damasceno Trevilato

**Affiliations:** I Hospital Moinhos de Vento. Porto Alegre, Rio Grande do Sul, Brazil

**Keywords:** Sterile Processing Department, Sustainable Development, Environmental Management, Environmental Responsibility, ISO 14000., Centro de Material y Esterilización, Desarrollo Sostenible, Gestión Ambiental, Responsabilidad Ambiental, ISO 14000.

## Abstract

**Objective:**

to report the experience of implementing the principles of ISO 14001 in the Sterile Processing Department of a large healthcare institution.

**Methods:**

a quantitative, descriptive experience report conducted in a large private hospital in the city of Porto Alegre, RS, Brazil, from January 2020 to July 2025.

**Results:**

the following initiatives were developed: rational use of water; optimization of surgical-grade paper consumption; and reorganization of sterile materials packaged individually. Educational actions, combined with the use of monitoring technologies, proved to be effective strategies for modifying behaviors and fostering an organizational culture focused on sustainability.

**Final considerations:**

the reported experience demonstrates how this approach can guide interventions and support sustainable decision-making, serving as a reference for organizations seeking to improve their environmental practices.

## RESUMEN

**Objetivo:**

informar sobre la experiencia de implementar los principios de la norma ISO 14001 en el Departamento Central de Suministro Estéril de una gran institución de salud.

**Métodos:**

se realizó un estudio cuantitativo, descriptivo e informativo de la experiencia en un gran hospital privado de Porto Alegre, RS, Brasil, desde enero de 2020 hasta julio de 2025.

**Resultados:**

se desarrollaron las siguientes iniciativas: uso racional del agua; optimización del consumo de papel quirúrgico; y reorganización de los materiales estériles envasados individualmente. Las iniciativas educativas, combinadas con el uso de tecnologías de monitoreo, revelaron estrategias eficaces para modificar comportamientos y fomentar una cultura organizacional centrada en la sostenibilidad.

**Consideraciones finales:**

la experiencia presentada demuestra cómo este enfoque puede orientar las intervenciones y respaldar la toma de decisiones sostenibles, sirviendo como referencia para las organizaciones que buscan mejorar sus prácticas ambientales.

## INTRODUCTION

The increasing complexity of environmental issues and global commitments to sustainable development have driven different sectors to adopt more robust and standardized environmental management systems (EMS), with ISO 14001 certification standing out in this context. ISO 14001 is an international standard that establishes requirements for implementing an EMS, enabling organizations to manage their environmental aspects and control their impacts, thereby consolidating itself as a strategic pillar for sustainability^(1)^.

The motivations for seeking ISO 14001 certification are diverse, ranging from the need to comply with regulatory requirements to the pursuit of competitive advantages in the market^(2,3)^. In addition, the adoption of EMS in hospital units promotes not only improvements in internal processes but also contributes to cultural and educational changes by increasing healthcare professionals’ awareness of the importance of sustainability^(3,4)^.

The implementation of ISO 14001 in healthcare institutions serves as a tool that enables organizations to improve their processes in ways that reduce environmental harm^(5)^. This approach contributes to the following Sustainable Development Goals (SDGs)^(6)^: Good Health and Well-Being (SDG 3); Clean Water and Sanitation (SDG 6); Responsible Consumption and Production (SDG 12); and Climate Action (SDG 13). In this sense, environmental certification is also positively associated with technical performance and economic efficiency in institutions, reinforcing the value of socio-environmental responsibility as a strategic differentiator^(7)^.

Particularly, the Sterile Processing Department (SPD) plays a fundamental role in this context, as it concentrates activities that involve significant consumption of natural resources and waste generation. Therefore, transforming this unit into a space that promotes educational practices and sustainability-oriented policies is essential for strengthening environmental management in healthcare institutions.

Despite the growing adoption of ISO 14001, there is still a notable lack of studies detailing its implementation and effects in the SPD. This gap highlights the need to deepen knowledge about the application of the standard’s principles in this specific setting, particularly regarding its role in promoting educational practices and sustainable policies within healthcare institutions.

## OBJECTIVE

To report the experience of implementing the principles of ISO 14001 in the SPD of a large healthcare institution.

## METHODS

### Ethical aspects

As this is an experience report, this study did not require approval by a Research Ethics Committee. However, institutional authorization was obtained for the dissemination of results.

### Study design, period, and setting

This is a quantitative, descriptive experience report. To ensure transparency, integrity, and methodological rigor in the manuscript, the recommendations of the STrengthening the Reporting of OBservational studies in Epidemiology (STROBE) checklist were followed. Although STROBE is primarily intended for observational studies, its guidelines were adapted for this report to ensure logical structuring of the data and clarity in the presentation of results.

The study was conducted from January 2020 to July 2025 in a large private hospital in Porto Alegre, RS, Brazil. The institution has 485 beds and 18 operating rooms, performing approximately 2,000 surgeries per month. The SPD is equipped with three washer-disinfectors, three ultrasonic devices, one dryer, three steam autoclaves, and two low-temperature hydrogen peroxide sterilizers. The unit operates in a centralized manner, processing an average of 50,000 items per month.

### Population or sample; inclusion and exclusion criteria

The study population consisted of institutional documentary records related to water consumption, the amount of surgical-grade paper waste discarded over a 24-hour period, and the indicator of reprocessing of items processed monthly.

The inclusion criteria comprised institutional records of water consumption from 2020 to 2025, surgical-grade paper waste generated during the preparation of individual instruments over a 24-hour period, and sterile materials whose expiration date had elapsed in the unit’s storage area.

The exclusion criteria comprised waste from other types of packaging (such as SMS or rigid containers), in order to maintain focus on the specific waste reduction action, as well as materials reprocessed for reasons unrelated to expiration (such as drops or packaging breaches), since the indicator specifically addressed inventory management.

### Study protocol

Data were obtained through monitoring processes and existing institutional indicators. Quantitative data on water consumption were extracted from institutional indicators provided by the environmental management team, which already consolidate continuous monitoring, including material consumption, waste disposal, and operational process monitoring. As a direct data collection instrument, a structured checklist was used to record the operational workflow.

### Analysis of results and statistics

Quantitative data were organized in spreadsheets and analyzed using simple descriptive statistics, summarizing performance based on monitored indicators and allowing the identification of patterns and trends. As this is an experience report, the data were used strictly for descriptive purposes, aiming to illustrate workflows and the volume of activities observed during the study period, without the intention of performing inferential statistical analyses, but rather to represent the described care context.

### Declaration on generative artificial intelligence and artificial intelligence-assisted technologies in the drafting process

During the preparation of this work, the authors used GPT-4o mini^®^ and Gemini 2.5^®^ Flash to improve clarity, coherence, and linguistic quality of the manuscript. After using these tools, the content was carefully reviewed and edited by the authors, who assume full responsibility for the final version of the manuscript. The figures were created with the support of generative artificial intelligence tools, Napkin IA^®^ and Gemini 2.5^®^ Flash.

## RESULTS

The implementation of ISO 14001 principles in the SPD enabled the structuring of a set of actions focused on environmental sustainability. The initiatives developed included rational water use, optimization of surgical-grade paper consumption, and reorganization of individually packaged sterile materials (Figure 1).

**Figure 1:**
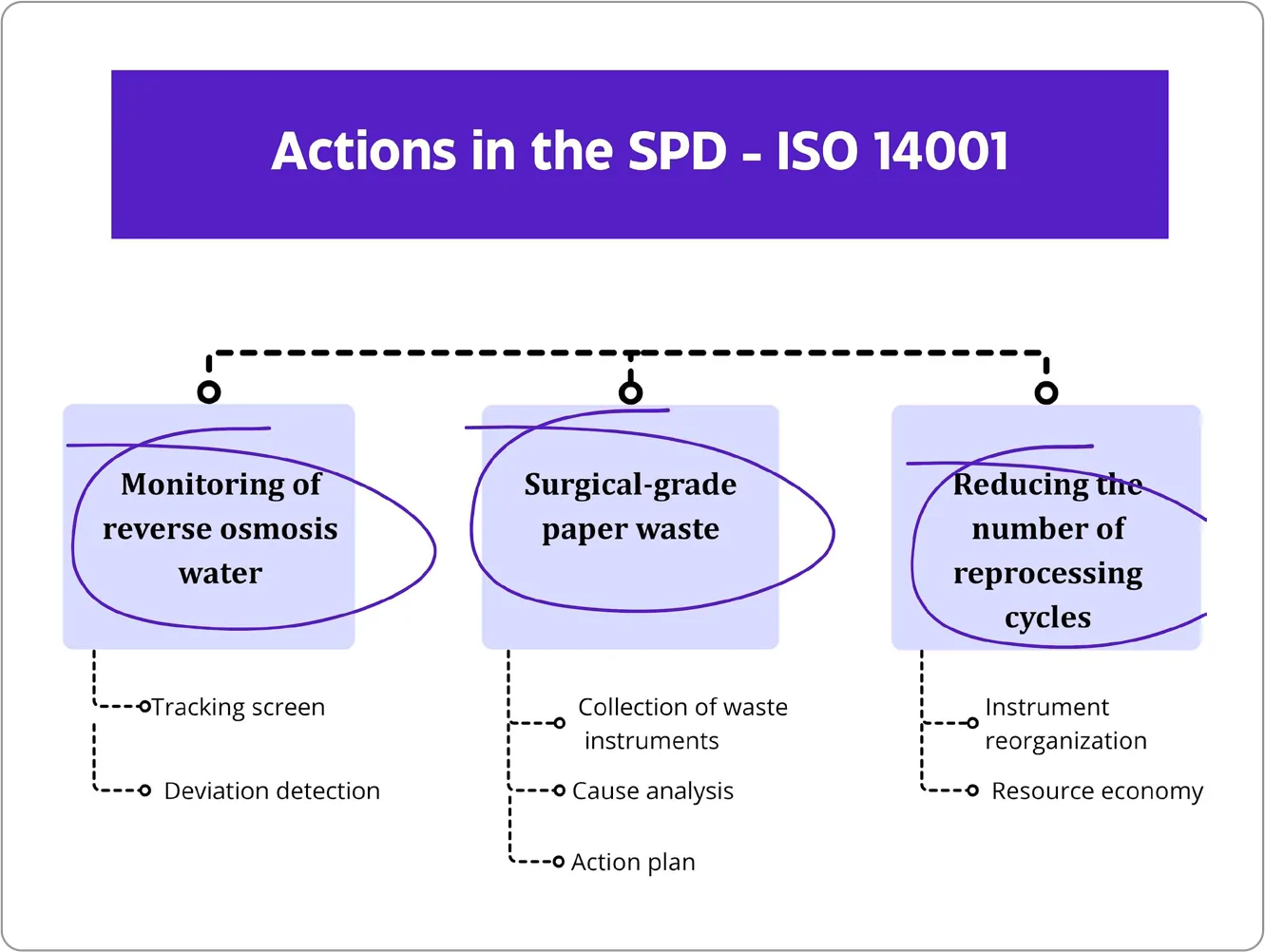
Actions developed in the Sterile Processing Department based on the principles of ISO 14001. Porto Alegre, Rio Grande do Sul, Brazil, 2025

### Action 1: Real-time monitoring of osmosis water consumption

After a period of low demand in 2021 and 2022 (Figure 2), the institution recorded an increase in consumption in 2023, directly reflecting full institutional occupancy (93-95%) in the post-pandemic period. The lack of control over reverse osmosis water was identified as a critical factor. To address this issue, in 2024, meters with real-time monitoring systems and strategic visualization within the SPD were implemented. This resource enabled continuous monitoring of water volumes and rapid correction of operational failures or leaks. The implemented initiative resulted in a reduction in water consumption across the institution, with the SPD being one of the sectors contributing to this outcome, given that water is used in all cleaning and sterilization processes.

**Figure 2:**
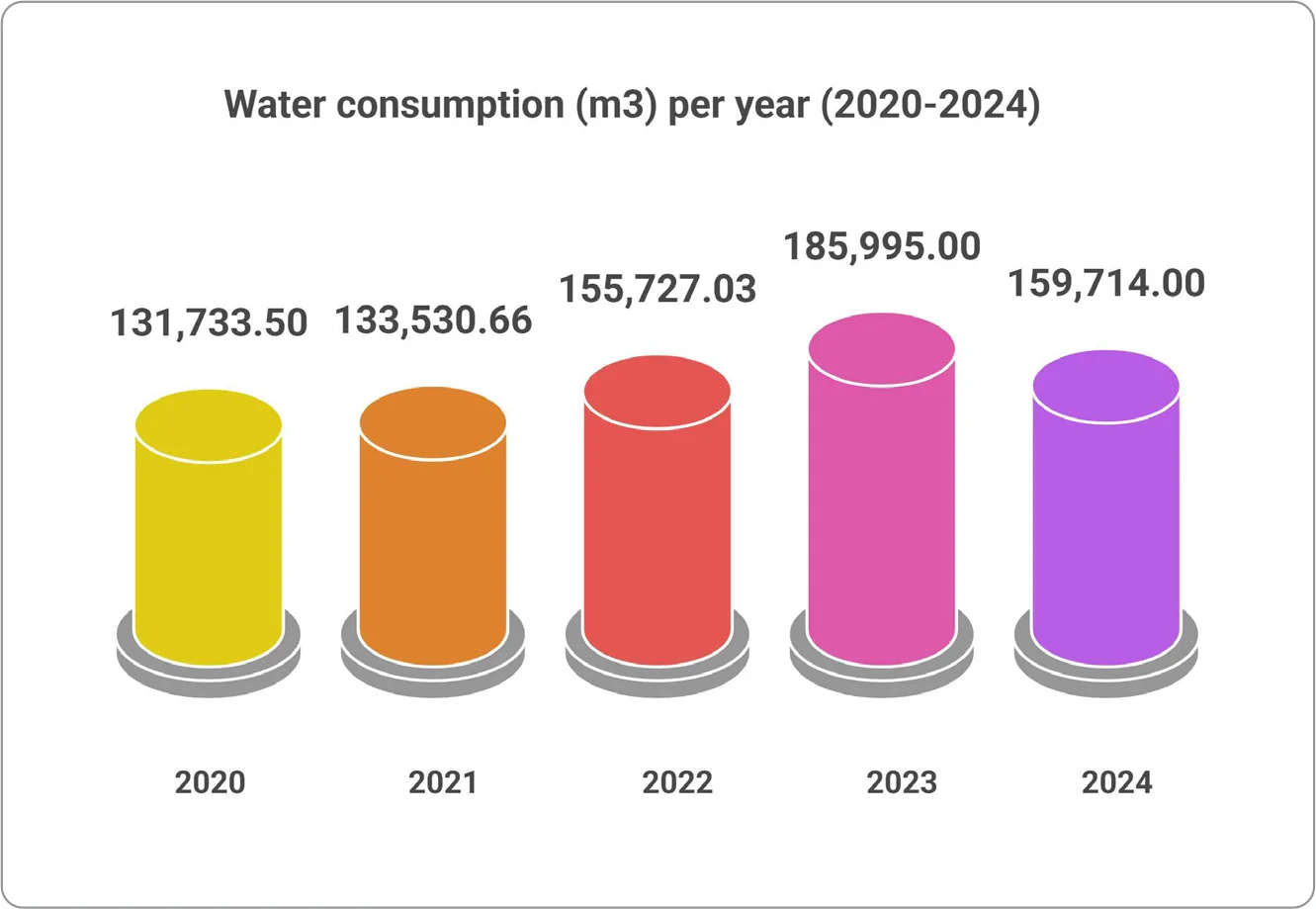
Total water consumption between 2020 and 2024. Porto Alegre, Rio Grande do Sul, Brazil, 2025

In 2025 (Figure 3), the institution reinforced its commitment to sustainability by launching the “Planetary Nursing” project on World Environment Day. This initiative encouraged the nursing team to adopt more conscious practices to reduce water and energy consumption, combat waste, and promote awareness of climate change.

**Figure 3:**
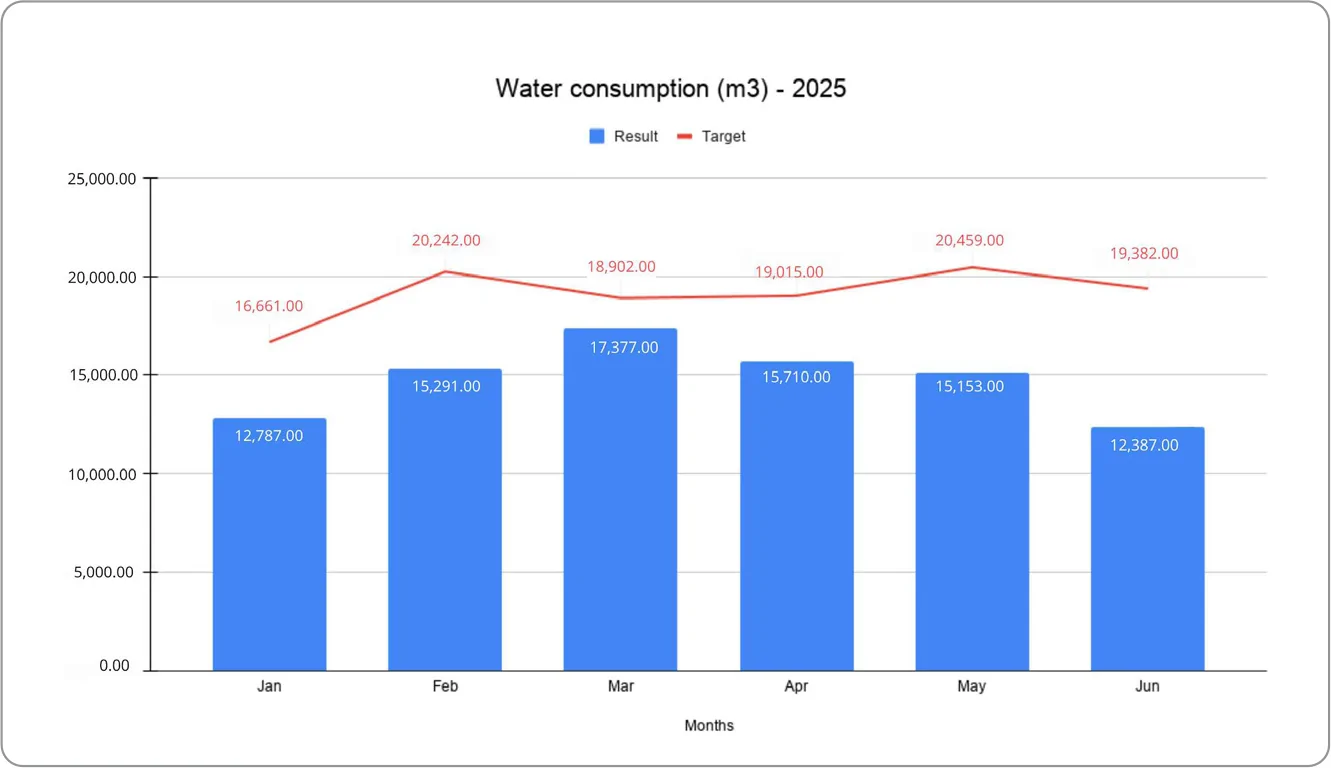
Total water consumption per month in 2025. Porto Alegre, Rio Grande do Sul, Brazil, 2025

### Action 2: Surgical-grade paper waste

Concerning the use of surgical-grade paper, a practical and educational initiative was implemented. This action consisted of the systematic collection of excess material and trimming waste generated during the preparation and packaging of individual surgical instruments, carried out continuously across all work shifts over a 24-hour period. During material preparation, the excess generated from adjusting packaging to instrument size was collected and measured, allowing the calculation of the total volume of avoidable waste, totaling 8.35 meters (Figure 4). The diversity of manufacturers and the absence of standardized visual parameters in packaging make precise cutting difficult for professionals. Therefore, data structuring focused on excess linear length (trimming waste), a universal variable independent of brand or material size. This approach made it possible to identify that waste occurs primarily at the sealing margin, regardless of instrument size.

**Figure 4:**
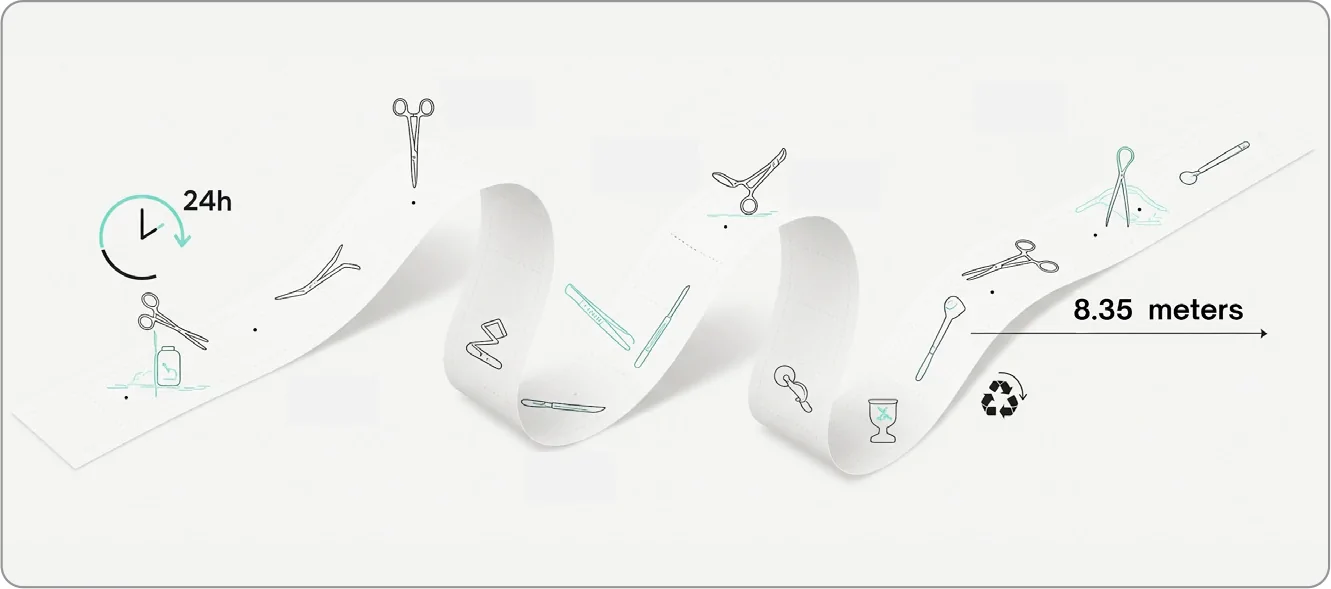
Surgical-grade paper scraps collected over 24 hours. Porto Alegre, Rio Grande do Sul, Brazil, 2025

**Figure 5:**
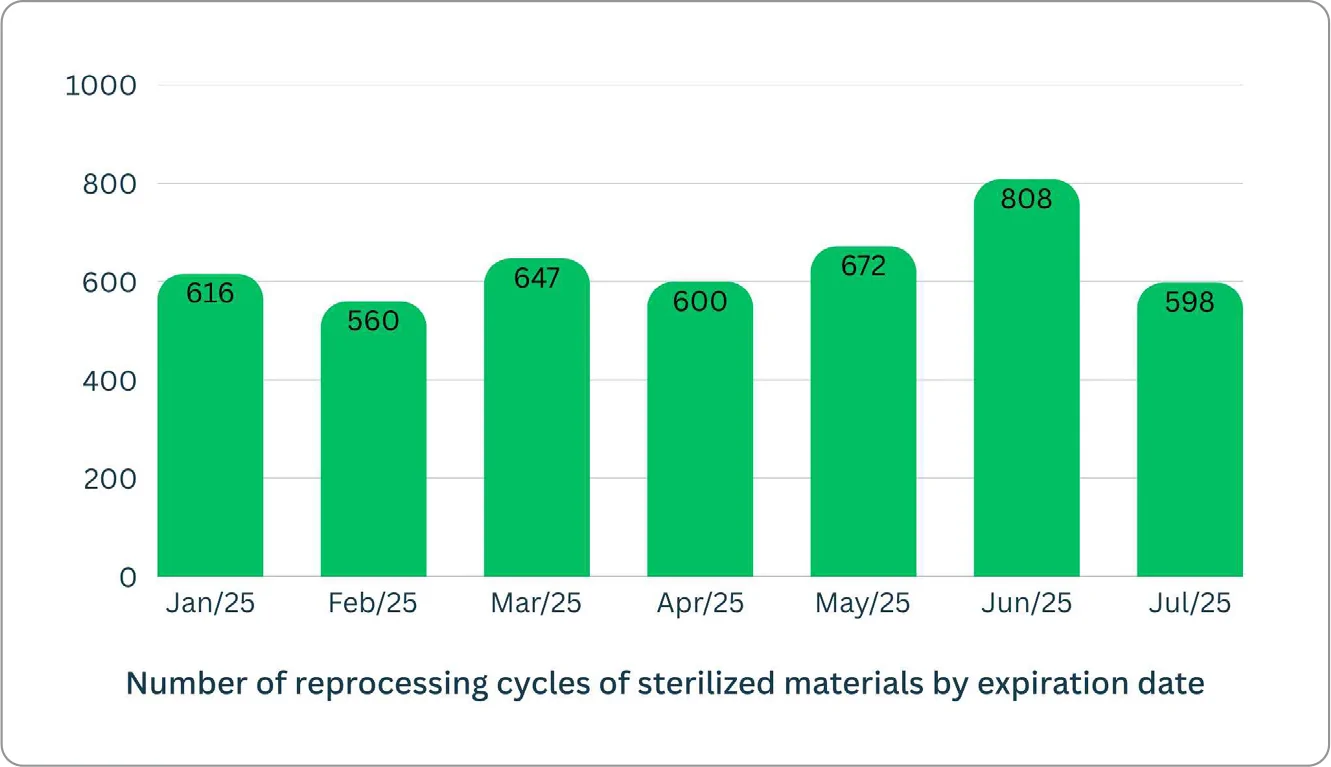
Number of reprocessing cycles of sterilized materials per unit. Porto Alegre, Rio Grande do Sul, Brazil, 2025

The main objective of this measure was to assess the amount of waste generated and identify opportunities to optimize the process. The results were shared with the entire team, along with guidance on the rational use of surgical-grade paper and the importance of conscious consumption. Although the collection was not converted into a formal monitoring indicator, the initiative was essential for raising awareness among staff, promoting reflection on the appropriate use of surgical-grade paper by adjusting packaging to the size of the instruments. In recognition of this initiative, the project received third place in the 2024 Eco Collaborator Award, an institutional event that highlighted it as an innovative and exemplary sustainability practice in hospital environments.

### Action 3: Reduction in the number of reprocessing cycles

The re-sterilization of individual materials due to expiration dates resulted in significant waste of time, energy, and packaging, in addition to causing wear on instruments. To improve monitoring and intervention in this process, an indicator-“number of reprocessing cycles of sterilized materials due to expiration”-was developed, enabling analysis of trends associated with reprocessing. As part of this monitoring process, the storage service conducts inspections divided by shifts and areas on weekends, auditing expiration dates according to the type of sterilization and anticipating the reprocessing flow of items due to expire in the following week. In addition to expiration dates, sealing quality, packaging integrity, and overall condition are also assessed.

Based on this initiative, a detailed inventory of individual materials was conducted, leading to grouping and assembly of new sets called “basic 1 trays”. With this strategy, it was possible to expand the inventory by eight new trays while simultaneously reducing 210 individual forceps per month that were previously prepared and processed separately. As a result, inventory management was optimized, the risk of expiration of sterile materials was reduced, and the consumption of supplies, energy, and labor time associated with the sterilization cycle decreased.

The three central initiatives of this study were based on the identification of environmental aspects and impacts, as recommended by ISO 14001, enabling strategic alignment with the United Nations SDGs:

- Osmosis water monitoring: through strict technological control, water waste was mitigated, addressing SDG 6 (Clean Water and Sanitation) and reinforcing SPD operational efficiency.
- Surgical-grade paper waste: optimization of packaging consumption directly impacted SDG 12 (Responsible Consumption and Production), reducing solid waste generation and associated costs.
- Reduction of re-sterilization due to expiration: by reducing unnecessary reprocessing, energy consumption and chemical inputs were minimized, contributing to climate protection (SDG 13) and ensuring patient safety, in line with SDG 3 (Good Health and Well-Being).

## DISCUSSION

The application of the standard contributed to fostering an organizational culture focused on environmental responsibility, efficient waste management, and strengthening the commitment to sustainable development in the hospital context. Although there are few studies specifically addressing the direct application of ISO 14001 in this setting, the implementation of its principles demonstrated positive results in strengthening sustainable practices, particularly in reducing resource consumption and improving materials management.

Real-time monitoring of osmosis water consumption provided greater operational control, enabling rapid identification of improper or excessive use. This finding is consistent with the literature, which highlights the role of technology as a facilitating element in hospital environmental management^(4)^. The observed reduction in water consumption suggests that integrating technology with environmental education can promote important cultural changes and consolidate sustainable practices within the sector. This initiative is directly aligned with SDG 6^(6)^, which aims to ensure water availability through sustainable management.

Similarly, measuring and presenting surgical-grade paper waste to the team encouraged important reflections on the rational use of supplies, reinforcing the educational potential of environmental management practices. Measuring waste over a 24-hour period served as a reference parameter, allowing the team to visualize the magnitude of waste at a larger scale. This result aligns with findings by Vicente *et al*. (2015)^(3)^, who highlight environmental certification as a tool capable of promoting cultural change in hospital units through awareness and continuing education. By transforming concrete data into learning tools, the SPD strengthened the sense of shared responsibility among staff and encouraged the adoption of more conscious and sustainable practices. This action directly contributes to SDG 12^(6)^, which aims to reduce waste generation and promote efficient resource use.

The development of a specific indicator for the reprocessing of sterile materials and the reorganization of the arsenal resulted in concrete efficiency gains, highlighting the importance of integrating environmental management and logistics tools in SPD. The reduction in individually processed forceps not only represents a numerical decrease but also impacts the lifespan of materials-by reducing wear from repeated cycles-and decreases autoclave energy consumption. These findings confirm that ISO 14001 certification supports organizational innovation and improves technical and operational efficiency^(8)^. Similarly, Robayo-Avendaño & Prato-García (2023)^(9)^ demonstrate that the adoption of management tools based on participatory methodologies can drive innovation, improve operational performance, and sustain organizational cultural transformation. These findings are consistent with the results observed in the SPD, reinforcing that well-planned and monitored initiatives enhance both operational efficiency and team engagement in sustainability practices. The results also corroborate Jabbar *et al*. (2025)^(10)^, who identified that successful ISO 14001 implementation depends on factors such as leadership engagement and resource commitment.

This reinforces that the improvements achieved in the SPD are not only the result of technical actions but are also supported by institutional governance that promotes innovation and environmental management. By reducing reprocessing, there is a consequent reduction in energy consumption and emissions associated with sterilization cycles, contributing to SDG 13_(6)_ for climate mitigation. This action is also aligned with SDG 3^(6)^, as improved management of sterile materials enhances safety and quality of care. Reducing the reprocessing of expired materials decreases the risk of process failures, promoting a safer hospital environment and contributing to the health and well-being of patients and professionals.

Beyond immediate benefits, this study highlights the need for continuity and expansion of these practices to ensure long-term sustainability and dissemination across other institutional sectors. Continuous monitoring and improvement support the establishment of environmental performance indicators and periodic process review to identify new opportunities for enhancement.

### Study limitations

The subjective nature of this study, combined with its conduction in a single institution, limits the reproducibility and generalizability of the results, as they are closely linked to the characteristics and resources of the local context. Additionally, team adherence and engagement may have been influenced by the specific institutional environment.

### Contributions to nursing, health, or public policy

This study provides relevant contributions to environmental management in healthcare services, particularly in the SPD context. It highlights the integration of technology, education, and environmental management as a strategy to reduce waste and improve operational efficiency. Furthermore, the reported experience reinforces the importance of systematic monitoring through indicators as a means of guiding interventions and supporting sustainable decision-making processes. Finally, it expands understanding of the potential of environmental management actions in hospital settings, strengthening the role of the SPD not only as a technical-operational unit but also as a promoter of an organizational culture focused on socio-environmental responsibility.

## FINAL CONSIDERATIONS

The reported experience demonstrated that implementing ISO 14001 principles in the SPD can act as a catalyst for promoting sustainable practices in the hospital environment. The results showed advances through systematic monitoring of osmosis water, team awareness regarding the rational use of surgical-grade paper, and optimization of inventory control, which reduced reprocessing due to expiration. Although the experience strengthened a culture of resource conservation, gaps remain, particularly regarding the need for quantitative assessment of the financial impact of these actions and analysis of the full life cycle of processed materials, in order to establish more robust environmental performance indicators.

Even in complex environments such as hospitals, it is possible to align technical efficiency with environmental and social commitments established by the SDGs. The SPD experience reinforces the strategic role of this unit as a space for innovation, education, and institutional transformation, extending beyond its traditional technical function. Thus, this study contributes to expanding understanding of the application of ISO 14001 in the healthcare sector, particularly in critical areas such as the SPD, and provides practical guidance for institutions seeking to adopt robust environmental management models aligned with contemporary demands for sustainability, efficiency, and social responsibility.

## Data Availability

The available research data is in the body of the article.
