# Supplementary material for: Environmental responsibility in the Sterile Processing Department: nursing leadership in sustainable development
Source: Rev Bras Enferm. 2026 Oct 16;79:e20250487. doi: 10.1590/0034-7167-2025-0487 (PMC13679162; doi:10.1590/0034-7167-2025-0487)
Supplement: Translation

# Translation: Responsabilidade ambiental no Centro de Material e Esterilização: o protagonismo da enfermagem no desenvolvimento sustentável

**DOI:** 10.1590/0034-7167-2025-0487pt

**Authors:** Renata Pereira Silva Artioli, Sidiclei Machado Carvalho, Maricel Silveira Corrêa, Denilse Damasceno Trevilato

**Affiliations:** I Hospital Moinhos de Vento. Porto Alegre, Rio Grande do Sul, Brasil

**Keywords:** Central de Esterilização e de Materiais, Desenvolvimento Sustentável, Gestão Ambiental, Responsabilidade Ambiental, ISO 14000.

## Abstract

**Objetivo:**

relatar a experiência da implementação dos princípios da norma ISO 14001 no Centro de Material e Esterilização de uma instituição de saúde de grande porte.

**Métodos:**

estudo quantitativo, descritivo, do tipo relato de experiência desenvolvido em um hospital privado de grande porte da cidade de Porto Alegre, RS, Brasil, no período de janeiro de 2020 a julho de 2025.

**Resultados:**

foram desenvolvidas as seguintes iniciativas: uso racional da água; otimização do consumo do papel grau cirúrgico; e reorganização de materiais estéreis embalados de forma unitária. Ações educativas, aliadas ao uso de tecnologias de monitoramento, revelaram estratégias eficazes para modificar comportamentos e despertar uma cultura organizacional voltada à sustentabilidade.

**Considerações finais:**

a experiência apresentada demonstra como essa abordagem pode direcionar intervenções e embasar a tomada de decisões sustentáveis, servindo como referência para organizações que buscam aprimorar suas práticas ambientais.

## INTRODUÇÃO

A crescente complexidade das questões ambientais e os compromissos globais com o desenvolvimento sustentável impulsionaram diferentes setores a adotar sistemas de gestão ambiental (SGAs) mais robustos e padronizados, destacando-se, nesse contexto, a certificação pela norma ISO 14001. Esta é uma norma internacional que estabelece requisitos para a implementação de um SGA, permitindo que as organizações gerenciem seus aspectos ambientais e controlem seus impactos, consolidando-se como um pilar estratégico para a sustentabilidade^(1)^.

As motivações para a busca pela certificação ISO 14001 são diversas, abrangendo desde a necessidade de atendimento até exigências regulatórias, além do objetivo de alcançar vantagens competitivas no mercado^(2,3)^. Além disso, a adoção de SGAs em unidades hospitalares promove não apenas melhorias nos processos internos, mas também contribui para mudanças culturais e educacionais, ao elevar a conscientização dos profissionais de saúde sobre a importância da sustentabilidade^(3,4)^.

A implementação da ISO 14001 em instituições hospitalares funciona como uma ferramenta que permite a organização melhorar seus processos de forma a torná-los menos prejudiciais ao meio ambiente^(5)^. Essa abordagem materializa contribuições para os seguintes Objetivos de Desenvolvimento Sustentável (ODS)^(6)^: Promover a Saúde e o Bem-Estar (ODS 3); Água Potável e Saneamento (ODS 6); Consumo e Produção Sustentáveis (ODS 12); e Ação Contra a Mudança Global do Clima (ODS 13). Nesse sentido, a certificação ambiental também se relaciona positivamente com o desempenho técnico e a eficiência econômica das instituições, reforçando o valor da responsabilidade socioambiental como um diferencial estratégico^(7)^.

Particularmente, o Centro de Material e Esterilização (CME) desempenha um papel fundamental dentro dessa perspectiva, uma vez que concentra atividades que envolvem significativo consumo de recursos naturais e geração de resíduos. Assim, sua transformação em um espaço promotor de práticas educativas e políticas voltadas à sustentabilidade é essencial para fortalecer a gestão ambiental nas instituições de saúde.

Mesmo com o aumento na adoção da norma ISO 14001, ainda há uma carência notável de estudos que detalham a implementação e os efeitos dessa certificação na CME. Essa lacuna evidencia a necessidade de aprofundar o conhecimento sobre a aplicação dos princípios da norma nesse ambiente específico, especialmente quanto ao seu papel na promoção de práticas educativas e políticas sustentáveis dentro das instituições de saúde.

## OBJETIVO

Relatar a experiência da implementação dos princípios da norma ISO 14001 no CME de uma instituição de saúde de grande porte.

## MÉTODOS

### Aspectos éticos

Por se tratar de relato de experiência, este estudo não exigiu a aprovação de um Comitê de Ética em Pesquisa. Contudo, obteve-se a anuência da instituição para divulgação dos resultados.

### Desenho, período e local do estudo

Trata-se de estudo quantitativo e descritivo do tipo relato de experiência. Para garantir a transparência, a integridade e o rigor metodológico na redação do manuscrito, seguiram-se as recomendações do *checklist STrengthening the Reporting of OBservational studies in Epidemiology* (STROBE). Embora o STROBE seja primordialmente destinado a estudos observacionais, suas diretrizes foram adaptadas para este relato visando à estruturação lógica dos dados e à clareza na descrição dos resultados.

O estudo foi desenvolvido no período de janeiro de 2020 a julho de 2025 em um hospital privado de grande porte da cidade de Porto Alegre, RS, Brasil. A instituição conta com 485 leitos e possui 18 salas cirúrgicas, onde são realizadas cerca de 2.000 cirurgias por mês. A CME conta com um parque tecnológico composto por três lavadoras termodesinfectadoras, três equipamentos ultrassônicos, uma secadora, três autoclaves a vapor e duas esterilizadoras de baixa temperatura por peróxido de hidrogênio. O setor opera de forma centralizada, com processamento de 50 mil itens em média por mês.

### População ou amostra; critérios de inclusão e exclusão

A população do estudo compreendeu os registros documentais institucionais com relação ao consumo de água, à quantidade de sobras de papel grau cirúrgico descartado em 24 horas e ao indicador de reprocessamento de itens processados mensalmente.

Os critérios de inclusão abrangeram registros institucionais de consumo de água no período de 2020 a 2025, sobras de papel grau cirúrgico geradas durante o preparo de instrumentais avulsos durante o período de 24 horas e materiais estéreis que tiveram sua validade expirada no arsenal da unidade.

Os critérios de exclusão incluíram sobras de outros tipos de embalagem que não o papel grau cirúrgico (como SMS ou contêineres rígidos), para manter o foco na ação de redução de desperdício específica, bem como materiais reprocessados por motivos alheios à validade (como queda no chão ou violação de embalagem), visto que o indicador foca na gestão do arsenal.

### Protocolo do estudo

Os dados foram obtidos a partir do acompanhamento dos processos e dos registros dos indicadores institucionais já existentes. Os dados quantitativos quanto ao consumo de água foram extraídos dos indicadores institucionais fornecidos pela equipe de gestão ambiental, que já consolidam o monitoramento contínuo, incluindo dados sobre consumo de materiais, descarte de resíduos e monitoramento de processos operacionais. Como instrumento de coleta direta, utilizou-se um roteiro de verificação estruturado para registrar o fluxo operacional.

### Análise dos resultados e estatística

Os dados quantitativos foram organizados em planilhas eletrônicas e analisados por meio de estatística descritiva simples, sintetizando o desempenho em relação aos indicadores monitorados, permitindo identificar padrões e tendências. Por se tratar de relato de experiência, esses dados foram utilizados de maneira estritamente descritiva, com o objetivo de elucidar os fluxos e o volume de atividades acompanhadas no período, sem a pretensão de fazer inferências estatísticas indutivas, mas visando demonstrar a realidade assistencial descrita.

### Declaração de inteligência artificial generativa e tecnologias assistidas por inteligência artificial no processo de redação

Durante a preparação deste trabalho, os autores usaram o GPT-4o mini^®^ e o Gemini 2.5^®^ flash para melhora da clareza, coesão e qualidade linguística do manuscrito. Depois de usar essas ferramentas/serviço, os conteúdos foram cuidadosamente revisados e editados pelos próprios autores, que assumem total responsabilidade pelo conteúdo da publicação. As ilustrações foram criadas com o auxílio da inteligência artificial generativa Napkin IA^®^ e do Gemini 2.5^®^ flash.

## RESULTADOS

A implementação dos princípios da norma ISO 14001 na CME possibilitou a estruturação de um conjunto de ações com foco na sustentabilidade ambiental. As iniciativas desenvolvidas envolveram uso racional da água, otimização do consumo do papel grau cirúrgico e reorganização de materiais estéreis embalados de forma unitária (Figura 1).

**Figura 1:** Ações desenvolvidas no Centro de Material e Esterilização baseadas nos princípios da ISO 14001. Porto Alegre, Rio Grande do Sul, Brasil, 2025

### Ação 1: Monitoramento do consumo da água de osmose em tempo real

Após o período de baixa demanda em 2021 e 2022 (Figura 2), a instituição registrou uma escalada no consumo em 2023, reflexo direto da plena ocupação institucional (93-95%) no pós-pandemia. Identificou-se que a ausência de controle sobre a água de osmose reversa era um fator crítico. Para mitigar o problema, em 2024, foram implementados medidores com sistema de monitoramento em tempo real e visualização estratégica na CME. O recurso possibilitou o acompanhamento contínuo dos volumes e a rápida correção de falhas operacionais ou vazamentos. A iniciativa implementada resultou em redução no volume de água utilizada em toda a instituição, sendo a CME um dos setores que pode ter contribuído para esse resultado, já que a água é utilizada em todos os seus processos de limpeza e esterilização.

**Figura 2:** Consumo de água total entre os anos de 2020 e 2024. Porto Alegre, Rio Grande do Sul, Brasil, 2025

Em 2025 (Figura 3), a instituição reforçou seu compromisso com a sustentabilidade ao lançar o projeto “Enfermagem Planetária” no Dia Mundial do Meio Ambiente. A iniciativa convida a equipe de enfermagem a adotar práticas mais conscientes para reduzir o consumo de água e energia, combatendo o desperdício e promovendo a conscientização sobre as mudanças climáticas.

**Figura 3:** Consumo total de água por mês em 2025. Porto Alegre, Rio Grande do Sul, Brasil, 2025

### Ação 2: Desperdício de papel grau cirúrgico

Em relação à utilização de papel de grau cirúrgico, implementou-se uma iniciativa prática e educativa. Essa ação consistiu na coleta sistemática de excedentes e aparas provenientes da preparação e da embalagem de materiais cirúrgicos avulsos, realizada de forma contínua em todos os turnos de trabalho, cobrindo o período de 24 horas. Durante o preparo dos materiais, as sobras provenientes do ajuste das embalagens ao tamanho dos instrumentos foram coletadas e medidas, permitindo calcular o volume total de descarte passível de mitigação, totalizando 8,35 metros (Figura 4). A diversidade de fabricantes e a ausência de parâmetros visuais padronizados nas embalagens dificultam a precisão do corte pelo profissional; por esse motivo, a estruturação dos dados focou no comprimento linear excedente (aparas), uma variável universal que independe da marca do insumo ou da dimensão do material embalado. Essa abordagem permitiu identificar que o desperdício ocorre primordialmente na margem de selagem, independentemente de ser um instrumental pequeno ou grande.

**Figura 4:** Sobras de papel grau cirúrgico coletadas durante 24 horas. Porto Alegre, Rio Grande do Sul, Brasil, 2025

**Figura 5:** Número de reprocessamento de materiais esterilizados por unidade. Porto Alegre, Rio Grande do Sul, Brasil, 2025

O objetivo principal desta medida foi avaliar o desperdício produzido e identificar oportunidades para otimizar o processo. O resultado foi compartilhado com toda a equipe, com orientações sobre o uso consciente do papel grau e a importância do uso consciente do insumo. Embora a coleta não tenha sido convertida em um indicador formal de acompanhamento, a iniciativa foi fundamental para a sensibilização do grupo, promovendo a reflexão sobre o uso consciente de papel grau cirúrgico adequando as embalagens ao tamanho dos materiais. Em reconhecimento a essa ação, o projeto recebeu o terceiro lugar no Prêmio Eco Colaborador de 2024, evento institucional que destacou a iniciativa como uma prática inovadora e exemplar de sustentabilidade no ambiente hospitalar.

### Ação 3: Redução do número de reprocessamento

A reesterilização de materiais avulsos, devido à expiração dos prazos de validade, resultava em desperdício significativo de tempo, energia e embalagens, além de causar o desgaste dos instrumentos. Para otimizar o monitoramento e a intervenção nesse processo, desenvolveu-se um indicador, “número de reprocessamento de materiais esterilizados por validade”, permitindo uma análise das tendências associadas ao reprocessamento. Como parte do controle do indicador de reprocessamento, o serviço de armazenamento realiza inspeções divididas por turnos e áreas aos finais de semana, auditando as validades de acordo com o tipo de esterilização e antecipando o fluxo de reprocessamento dos itens com vencimento na semana subsequente. Além da data de validade, também são avaliados a qualidade da selagem, a integridade e o aspecto da embalagem.

A partir dessa iniciativa, foi realizado um levantamento detalhado dos materiais avulsos, com agrupamento e montagem de novas bandejas denominadas “básica 1”. Com essa estratégia, foi possível aumentar o arsenal em oito novas bandejas e, simultaneamente, promover uma redução de 210 unidades de pinças avulsas no mês que, anteriormente, eram preparadas e processadas individualmente. Dessa forma, a gestão do estoque é otimizada, o risco de vencimento de materiais estéreis diminui e o consumo de insumos, energia e tempo de trabalho associado ao ciclo de esterilização é reduzido.

As três iniciativas centrais deste estudo foram fundamentadas na identificação de aspectos e impactos ambientais, conforme preconiza a ISO 14001, permitindo o alinhamento estratégico com os ODS da ONU:

- Monitoramento da água de osmose: através do controle rigoroso e tecnológico, mitigou-se o desperdício hídrico, atendendo ao ODS 6 (Água Potável e Saneamento) e reforçando a eficiência operacional da CME.
- Desperdício de papel grau cirúrgico: a otimização do consumo de embalagens impactou diretamente o ODS 12 (Consumo e Produção Responsáveis), reduzindo a geração de resíduos sólidos e os custos associados.
- Redução de reesterilizações por vencimento: ao diminuir o reprocessamento desnecessário, reduziu-se o consumo de energia e insumos químicos, contribuindo para a proteção do clima (ODS 13) e garantindo a segurança do paciente, o que corrobora o ODS 3.

## DISCUSSÃO

A aplicação da norma contribuiu para o despertar de uma cultura organizacional voltada à responsabilidade ambiental, à gestão eficiente de resíduos e ao fortalecimento do compromisso com o desenvolvimento sustentável no contexto hospitalar. Embora não haja muitos estudos específicos sobre a aplicação direta da ISO 14001 nesse setor específico, a implementação dos princípios dessa norma internacional demonstrou resultados positivos no fortalecimento de práticas sustentáveis, especialmente no que se refere à redução do consumo de recursos e ao aprimoramento da gestão de materiais.

O monitoramento em tempo real do consumo de água osmótica proporcionou maior controle operacional, permitindo a identificação rápida de usos indevidos ou excessivos, o que corrobora a literatura, que destaca o papel da tecnologia como elemento facilitador na gestão ambiental hospitalar^(4)^. A redução no consumo de água observada sugere que a integração da tecnologia com a educação ambiental pode favorecer mudanças culturais importantes e consolidar práticas sustentáveis no setor. Essa iniciativa está diretamente alinhada ao ODS 6^(6)^, que busca garantir a disponibilidade através da gestão sustentável da água.

De modo semelhante, a ação de medição e exposição do desperdício de papel grau cirúrgico à equipe estimulou reflexões importantes sobre o uso racional dos insumos, reforçando o potencial educativo das práticas de gestão ambiental. A medição de sobras em apenas 24 horas serviu como um parâmetro de referência. Ao projetar esse dado, a equipe pôde visualizar a magnitude do desperdício em escala industrial. Esse resultado converge com as observações de Vicente, Suárez-Varela, Monrós e Gonzále (2015)^(3)^, que apontam a certificação ambiental como um instrumento capaz de promover mudanças culturais em unidades hospitalares por meio da sensibilização e da educação continuada das equipes. Ao converter informações concretas em ferramentas de aprendizagem, a CME fortaleceu o senso de corresponsabilidade entre os colaboradores e incentivou a adoção de práticas mais conscientes e sustentáveis. Essa prática contribui diretamente para o ODS 12^(6)^, que tem como meta a redução da geração de resíduos e a promoção do uso eficiente de recursos.

O desenvolvimento de um indicador específico para o reprocessamento de materiais estéreis e a reorganização do arsenal resultaram em ganhos concretos de eficiência, evidenciando a importância de integrar ferramentas de gestão ambiental e logística na CME. A otimização do arsenal com redução de pinças avulsas processadas individualmente não é apenas um número, mas uma variável que impacta a vida útil dos materiais (reduzindo o desgaste por ciclos repetitivos) e o consumo de energia da autoclave. Tais resultados ratificam que a certificação ISO 14001 favorece inovações organizacionais e melhora a eficiência técnica e operacional^(8)^. De maneira similar, Robayo-Avendaño & Prato-García (2023)^(9)^ demonstram que a adoção de ferramentas gerenciais específicas, quando fundamentadas em metodologias participativas e adaptadas às necessidades do setor, pode impulsionar processos inovadores, melhorar o desempenho operacional e sustentar transformações culturais organizacionais. Esses achados convergem com os resultados obtidos na CME, evidenciando que iniciativas bem planejadas e monitoradas elevam tanto a eficiência operacional quanto o engajamento das equipes no contexto da sustentabilidade. Os resultados também corroboram as conclusões de Jabbar *et al*. (2025)^(10)^, que, em sua análise de hospitais no Iraque, identificaram que o sucesso na implementação da ISO 14001 depende de fatores críticos, como o envolvimento da alta liderança e o comprometimento de recursos.

Isso reforça a ideia de que as melhorias obtidas na CME, além de serem resultado de ações técnicas bem planejadas, são sustentadas por uma governança institucional que apoia a inovação e a gestão ambiental, o que é fundamental para a efetividade dessas práticas a longo prazo. Ao reduzir a quantidade de reprocessamento, há uma consequente redução no consumo de energia e nas emissões de gases associadas a cada ciclo de reprocessamento, contribuindo com o ODS 13^(6)^ para a mitigação dos impactos climáticos. A referida ação também está alinhada com o ODS 3^(6)^, pois a gestão mais eficiente dos materiais estéreis aumenta a segurança e a qualidade do processo assistencial. Ao diminuir o reprocessamento de materiais vencidos, o risco de falhas no processo é reduzido, promovendo um ambiente hospitalar mais seguro, contribuindo, como consequência, para a saúde e o bem-estar dos pacientes e profissionais.

Além dos benefícios imediatos, este estudo reforça a necessidade de continuidade e expansão dessas práticas, de modo a garantir sua sustentabilidade ao longo do tempo, bem como sua disseminação para outros setores da instituição. O monitoramento e a melhoria contínua favorecem o estabelecimento de indicadores de desempenho ambiental e revisão periódica dos processos para identificar oportunidades de melhoria.

### Limitações do estudo

Considera-se que a subjetividade inerente ao estudo, somada à sua realização em uma única instituição, limita a reprodutibilidade e a generalização dos resultados, encontrando-se estritamente vinculados às características e recursos do cenário local. Ademais, a adesão e o engajamento da equipe podem ter sido influenciados pelo contexto institucional específico.

### Contribuições para a área da enfermagem, saúde ou políticas públicas

Este estudo oferece contribuições relevantes para a gestão ambiental em serviços de saúde, especialmente no contexto da CME, com integração entre tecnologia, educação e gestão ambiental como estratégia para a redução de desperdícios e a melhoria da eficiência operacional. Além disso, a experiência relatada reforça a importância de monitoramento sistemático através de indicadores como forma de orientar intervenções e apoiar processos de tomada de decisões sustentáveis, servindo como inspiração para outras instituições. Por fim, a prática descrita amplia a compreensão sobre o potencial das ações de gestão ambiental no ambiente hospitalar, fortalecendo a CME como espaço não apenas técnico-operacional, mas também promotor de cultura organizacional voltada para a responsabilidade socioambiental.

## CONSIDERAÇÕES FINAIS

A experiência relatada demonstrou que a implementação dos princípios da norma ISO 14001 na CME pode atuar como um catalisador para a promoção de práticas sustentáveis no ambiente hospitalar. Os resultados evidenciaram avanços através do monitoramento sistemático da água de osmose, da sensibilização da equipe sobre o uso racional do papel grau cirúrgico e da otimização do controle do arsenal, o que reduziu o reprocessamento por vencimento de esterilização. Embora a experiência tenha fortalecido a cultura de preservação de insumos, identificam-se como lacunas para aprofundamento a necessidade de mensuração quantitativa do impacto financeiro dessas ações e a análise do ciclo de vida total dos materiais processados, visando consolidar indicadores de desempenho ambiental ainda mais robustos para a instituição.

Mesmo em contextos complexos como o hospitalar, é possível alinhar a busca pela eficiência técnica com os compromissos ambientais e sociais preconizados pelos ODS. A experiência da CME reforça ainda o papel estratégico desse setor como ambiente de inovação, educação e transformação institucional, indo além de sua função técnica tradicional. Assim, este estudo contribui para ampliar a compreensão sobre as possibilidades de aplicação da norma ISO 14001 no setor da saúde, especialmente em áreas críticas, como a CME, e oferece subsídios práticos para outras instituições que desejam adotar modelos robustos de gestão ambiental, alinhados às demandas contemporâneas por sustentabilidade, eficiência e responsabilidade social.

## Data Availability

Os dados de pesquisa disponíveis estão no corpo do artigo.
